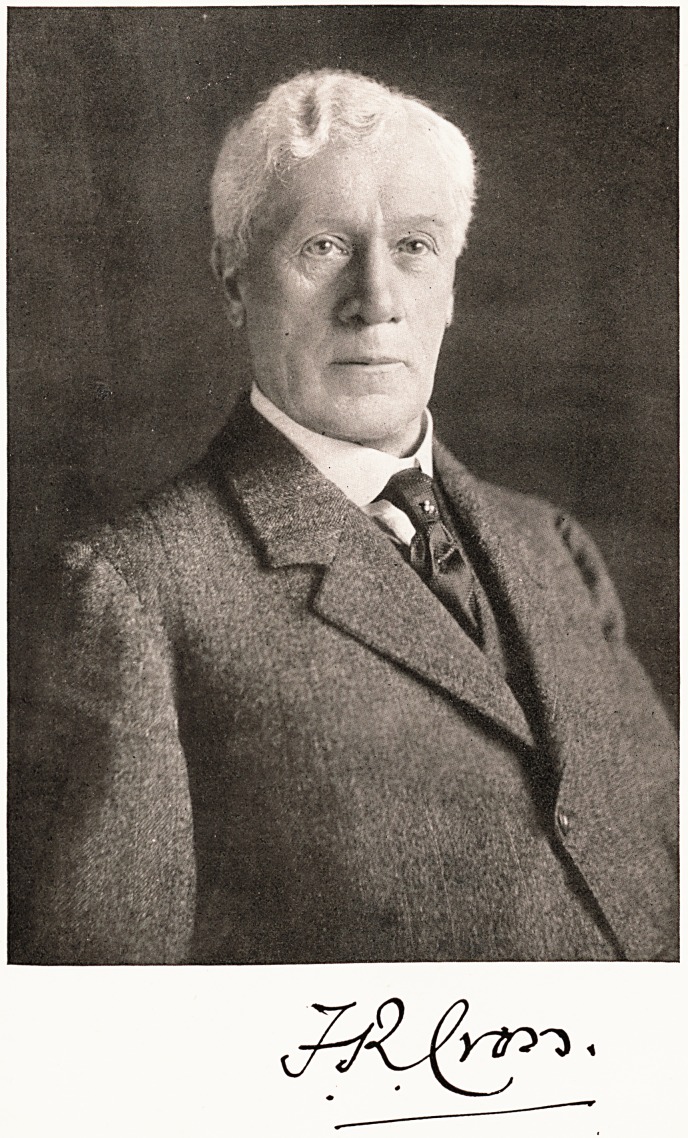# Francis Richardson Cross

**Published:** 1931

**Authors:** 


### Obituary

### FRANCIS RICHARDSON CROSS, M.B. Lond., F.R.C.S. Eng., LL.D. Bristol, J.P.

The death of Mr. Cross in his eighty-third year, on 12th July, brought to a close a life of strenuous work and noble enterprise. He was the eldest son of the Rev. Joseph Cross, Vicar of Merriott in South Somerset and formerly Precentor of Bristol Cathedral. He was educated at Crewkerne Grammar School and King's College Hospital, London. He took his Fellowship of the Royal College of Surgeons in 1878, and his M.B. (London) in 1879. He also attended clinics in Vienna, Berlin, Paris and Utrecht, and for some time worked as Clinical Assistant at Moorfields Eye Hospital. At King's College Hospital he held the posts of House Surgeon, Medical Tutor, and Sub-Dean.

Whilst working in London he became well known in the athletic world, winning the 100 yards in the inter-hospital sports in record time, and, we have been told, holding for that distance the amateur record for the year, a feat to which he sometimes referred with very natural pride.

It was in 1878 that he was invited to come to University College, Bristol, as Lecturer in Anatomy, and in the same year he was appointed Assistant Surgeon to the Bristol Royal Infirmary. A year later he became full surgeon, and in 1880 he was appointed Dean of the Faculty of Medicine at University College. Thus, to use his own expression, he had not been long in Bristol before he "got pitchforked into a lot of work."

Very shortly after this he turned his attention specially to ophthalmology. He became a member of the newly-formed Ophthalmological Society in 1881, and was a frequent contributor to its *Transactions*. In 1882 he was appointed Surgeon to the Bristol Eye Hospital. This institution, founded in 1810, had lapsed into a somewhat torpid state. Cross's energy and ability quickly made themselves felt. He soon became so busy both with his private and hospital work that he decided to give up general surgery. In 1885 he was appointed Ophthalmic Surgeon to the Bristol Royal Infirmary, a post

C. F. Cross.

which he occupied till 1900. Whilst studying in Utrecht Cross became friends with Professor Snellen, then one of the leading ophthalmic surgeons in Europe, and it was his son, the late Professor Herman Snellen, who in 1889 was appointed the first House Surgeon to the Bristol Eye Hospital.

In 1891 Cross was elected President of the Bristol Medico-Chirurgical Society. The subject of his presidential address was "Progress in Medicine; local and general," in which he stressed the need for special departments in all large hospitals. Amongst other varied and distinguished positions he held may be mentioned Lecturer, afterwards Reader, in Ophthalmology in the University of Bristol and President of the Ophthalmic Section at the British Medical Association meeting in Bristol in 1894. In 1898 he was elected a Member of the Council of the Royal College of Surgeons, and at the same time was Sheriff of Bristol. He delivered the annual oration at the Medical Society of London in 1901, the Bradshaw Lecture at the Royal College of Surgeons in 1909, the Long Fox Lecture in Bristol in 1915, and the Doyne Memorial Lecture at the Oxford Ophthalmological Congress in 1920. Besides all these engagements he was President of the Grateful Society in 1889, of the Dolphin Society in 1912, and of the University Colston Society in 1916, and was appointed a Magistrate in 1902. In 1914 he was elected President of the Ophthalmological Society of the United Kingdom, and many will recall the admirable way in which he conducted the meetings—courteous, friendly, alert and sympathetic; in every respect an ideal chairman.

After the tragically sudden death of his wife in 1920 the inevitable anxiety of active practice seemed to weigh more heavily, and to some extent he gave up operating—not that his skill was failing, but because he became unduly concerned about his patients, even though they might be making a perfect recovery. In 1925 he resigned his appointment as Surgeon to the Eye Hospital after more than forty-three years strenuous work. One of the last operations he performed there, shortly before his resignation, was the formation of an artificial pupil in a case of widely adherent leucoma. The case was a difficult one with an extremely shallow anterior chamber. He used a keratome and a Tyrrell's hook and performed the operation with the skill and precision of a man in his prime. He had always been a quick and resourceful operator, perhaps lacking the extreme delicacy of manipulation shown by some few of the masters of the art, but he was exceptionally successful. At one time he was a strong advocate of the removal of the lens in patients suffering from a high degree of myopia, and his results were very good, but in later years he rarely resorted to this operation. He was ready to undertake most of the newly-suggested operations, and it is rather remarkable, therefore, that he never adopted the operation of trephining for glaucoma.

Cross was a lover of the country and of country life, and for many years hunted with the Berkeley and the Duke of Beaufort's hounds, but in his busy life he had little leisure to indulge in these pursuits; his thoughts were always centred on his work or his civic and social duties. He had nothing but praise and admiration for research, but had himself no time to devote to it. When time allowed he studied ophthalmic literature, and he had a happy knack of picking out and using what was of practical value.

In various educational movements in Bristol he took an active part. For many years he was on the Council of the University and of Clifton College. He also was deeply interested in the cathedral and its services. In 1921 he joined with Dean Burroughs in initiating the annual service for the medical profession, held on the Sunday nearest to St. Luke's Day.

Like many other ophthalmic surgeons, Cross was alive to the lamentable wastage of sight owing to ophthalmia neonatorum and other preventable causes. From the first he took an active interest in the welfare of the blind. In 1901 he joined the Committee of the Bristol Blind Asylum (the Royal School of Industry for the Blind), then situated at the top of Park Street on the site of the present University buildings. He was largely instrumental in the extension and development of that Institution and the removal of the school to its present comparatively rural position at Westbury-on-Trym. He became Chairman of the Committee in 1913, and was elected President in 1921. One of his last activities in public life was at the end of September, 1930, when the Duchess of Beaufort opened Southmead House as a hostel for the senior boys at the school. The ceremony took place in the garden ; and none of those present on that sunny autumn afternoon who heard his interesting résumé of the history of the Blind Asylum, will readily forget his fine, upright figure, the wavy silver-white hair, the clear and pleasant voice, and the confident self-possession which was always his characteristic as a speaker.

Almost to the very last he took an active interest in the affairs of the Bristol Eye Hospital. He attended the monthly meeting of the committee shortly before his death. His portrait, painted by Miss B. Bright in 1920, is in the senate room at the University, and a replica of it hangs in the hall of the Eye Hospital, the institution which he rescued from obscurity, and to which he devoted so large a portion of his life. It is here, where he was so well known and valued, that he will, perhaps, be longest remembered; and where, when those who owed to him so much are long since dead, his example and his name will live.

## Figures and Tables

**Figure f1:**